# 5-Aminolevulinic Acid-Based Fluorescence Guidance in Urologic Oncology: Current Status, Pitfalls, and Future Directions

**DOI:** 10.3390/life16040546

**Published:** 2026-03-26

**Authors:** Takashi Matsuoka, Atsushi Igarashi, Toshinari Yamasaki, Mutsushi Kawakita

**Affiliations:** Department of Urology, Kobe City Medical Center General Hospital, Kobe 650-0047, Hyogo, Japan

**Keywords:** 5-aminolevulinic acid, photodynamic diagnosis, protoporphyrin IX, fluorescence-guided surgery, bladder cancer, renal cancer, partial nephrectomy

## Abstract

5-Aminolevulinic acid (5-ALA) induces tumor-selective accumulation of protoporphyrin IX (PpIX), enabling fluorescence-guided visualization of malignant tissue. In urologic oncology, the most established application is photodynamic diagnosis (PDD) during transurethral resection of non-muscle-invasive bladder cancer, in which fluorescence can identify occult carcinoma in situ and additional papillary lesions; however, specificity may decline in the presence of inflammation, recent instrumentation, or intravesical therapy. Renal applications are emerging: oral 5-ALA before partial nephrectomy can highlight some renal tumors, but fluorescence is often heterogeneous, can overlap with normal parenchyma, and is affected by histologic subtype, necrosis, blood attenuation, and device-dependent optics. Evidence in upper tract urothelial carcinoma and prostate cancer remains preliminary, with small cohorts and practical challenges in endoscopic or robotic workflows, alongside systemic adverse events such as hypotension and photosensitivity. This review synthesizes clinical and preclinical studies of 5-ALA-based fluorescence guidance across bladder, kidney, upper tract, and prostate malignancies, focusing on where the technology is ready for practice versus where it remains investigational. We discuss common pitfalls in interpretation and implementation and outline future directions, including quantitative fluorescence and spectroscopy, standardized dosing and imaging protocols, and prospective multicenter trials linking fluorescence guidance to residual disease, recurrence, margin status, and patient-centered outcomes.

## 1. Introduction

Accurate intraoperative visualization remains a major unmet clinical need in urologic oncology. In non-muscle-invasive bladder cancer (NMIBC), early recurrence after transurethral resection of bladder tumor (TURBT) remains common, despite advances in risk stratification and intravesical therapy. Contemporary international guidelines emphasize the importance of complete resection and sensitive detection of flat lesions, particularly carcinoma in situ (CIS), to reduce residual disease and subsequent recurrence [1,2]. Similar challenges exist in minimally invasive renal surgery, where limited tactile feedback and heterogeneous tumor margins complicate accurate assessment of complete excision.

**Figure 1:**
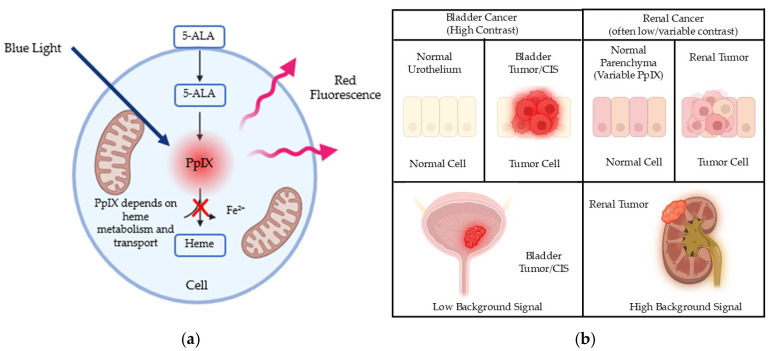
Conceptual framework of 5-ALA induced PpIX fluorescence guidance in urologic oncology. (**a**) After administration, 5-ALA enters cells and is metabolized through the heme biosynthesis pathway to generate PpIX, which can be converted to heme by FECH in an iron-dependent manner. PpIX emits red fluorescence under blue-violet excitation, enabling PDD. PpIX accumulation and tumor-to-normal contrast are influenced by multiple biological factors, including heme pathway activity, iron availability, and porphyrin transporters (for example, ABCG2-mediated efflux), as well as optical factors such as tissue attenuation and background autofluorescence. In the schematic, blue arrows indicate excitation light, red arrows indicate emitted fluorescence, and the red cross denotes inhibition of PpIX-to-heme conversion. (**b**) Typical site-dependent contrast patterns in urologic oncology. In bladder cancer, fluorescence contrast is often favorable for detecting carcinoma in situ and small or multifocal lesions, whereas specificity may decline in inflammatory or post-treatment settings. In renal tumors, fluorescence can be low or heterogeneous and may overlap with surrounding renal parenchyma, limiting margin visualization in some cases. Abbreviations: 5-ALA, 5-aminolevulinic acid; PpIX, protoporphyrin IX; PDD, photodynamic diagnosis; FECH, ferrochelatase; ABCG2, ATP-binding cassette subfamily G member 2. Created in BioRender. Matsuoka, T. (2026) https://BioRender.com/f9cmhoe.

5-Aminolevulinic acid (5-ALA) is an endogenous precursor in the heme biosynthesis pathway. Exogenous administration of 5-ALA leads to intracellular accumulation of protoporphyrin IX (PpIX), a photodynamically active fluorophore that emits red fluorescence at approximately 635 nm when excited by blue-violet light around 400–410 nm [3,4]. This principle underlies photodynamic diagnosis (PDD) and photodynamic therapy. The clinical feasibility of 5-ALA-guided surgery has been firmly established in malignant glioma, where a randomized phase III trial demonstrated improved extent of resection [5]. Pharmacokinetic studies further indicate that after systemic administration, 5-ALA and porphyrins show prominent renal handling with measurable urinary excretion, supporting translation to urologic applications [6]. Importantly, clinical fluorescence contrast depends primarily on tumor-selective intracellular PpIX accumulation relative to background autofluorescence, rather than on luminal exposure alone [7,8]. To provide an overview of the 5-ALA–PpIX fluorescence mechanism and the typical contrast differences across urologic sites, a simplified schematic is shown in Figure 1.

Tumor-selective PpIX accumulation is not universal and reflects tumor-specific regulation of heme metabolism and porphyrin transport. PpIX is normally converted to non-fluorescent heme by the mitochondrial enzyme ferrochelatase (FECH). Reduced FECH activity, together with altered uptake and efflux mechanisms, promotes intracellular PpIX accumulation in malignant cells [7,8]. Iron availability critically modulates this process, as iron serves as the substrate for FECH-mediated heme synthesis. Experimental studies have shown that manipulation of iron metabolism can markedly influence the specificity of 5-ALA-based photodynamic effects [9]. In renal cell carcinoma (RCC), variability in FECH activity and the PpIX efflux transporter ABCG2 contributes to heterogeneous fluorescence, and pharmacologic strategies targeting these pathways have been explored to enhance tumor contrast [10,11].

In bladder cancer, multiple prospective and real-world studies have consistently demonstrated that 5-ALA-PDD cystoscopy improves tumor detection compared with white light, including superior identification of CIS and multifocal lesions with either oral or intravesical administration [12,13,14]. Beyond diagnostic accuracy, accumulating clinical evidence indicates that PDD-assisted TURBT can reduce residual tumor and intravesical recurrence, suggesting a potential therapeutic benefit [15,16,17,18]. In contrast, translation to renal surgery has been less consistent. Although high sensitivity was reported in fluorescence-guided laparoscopic partial nephrectomy, subsequent robot-assisted series have demonstrated inconsistent intraoperative fluorescence and substantial background signals in normal renal parenchyma [19,20]. These organ-specific differences highlight both the promise and current limitations of 5-ALA-guided surgery in urologic oncology, underscoring the need for critical appraisal of its current status, pitfalls, and future directions.

At the same time, important knowledge gaps remain. Outside the most established non-muscle-invasive bladder cancer setting, standardized imaging and interpretation protocols for 5-ALA-guided procedures remain incompletely defined, and reported outcomes are difficult to compare across tumor types, administration routes, optical platforms, and surgical workflows. In upper tract urothelial carcinoma, the available evidence is still based largely on small single-center series with limited harmonization of lesion mapping and interpretation criteria; in renal cell carcinoma and prostate cancer, fluorescence findings remain more heterogeneous, and no standard-of-care role has been established. In addition, although perioperative hypotension and other adverse events associated with oral 5-ALA are increasingly recognized, risk stratification and mitigation strategies are still evolving and have not yet been uniformly validated across centers. A clearer articulation of these gaps is essential to determine where 5-ALA-guided imaging is ready for practice and where further prospective multicenter validation is required.

Recent studies have expanded the clinical evidence base for 5-ALA-guided approaches in urothelial cancers, including not only lesion detection and recurrence-related outcomes in non-muscle-invasive bladder cancer but also perioperative safety considerations associated with oral administration and emerging ureteroscopic applications in upper tract urothelial carcinoma. By contrast, contemporary renal surgery studies have highlighted heterogeneous fluorescence, background signal in normal renal parenchyma, and procedure-dependent limitations, suggesting that the clinical utility of 5-ALA is highly disease- and platform-specific. These divergent developments create a timely need for an updated review that clearly distinguishes established indications from investigational or currently constrained applications.

The primary objective of this narrative review is to critically evaluate the current status, clinical applications, limitations, and future prospects of 5-ALA-based fluorescence guidance in urologic oncology. Specifically, we ask which urologic malignancies currently benefit from 5-ALA-induced protoporphyrin IX fluorescence, what biological, technical, and safety-related factors limit its interpretation and implementation, and what developments are required for broader clinical translation. We therefore synthesize clinical and preclinical evidence across bladder cancer, upper tract urothelial carcinoma, renal cell carcinoma, and prostate cancer, focusing on where 5-ALA-guided imaging is ready for practice and where it remains investigational. For transparency, the literature included in this narrative review was identified through PubMed searches supplemented by manual screening of the reference lists of relevant review articles and primary studies. The search focused primarily on articles published from 2010 onward, although earlier landmark studies were also included when considered important for historical or clinical context. Search terms included “5-aminolevulinic acid”, “5-ALA”, “hexaminolevulinate”, “HAL”, “photodynamic diagnosis”, “fluorescence-guided surgery”, and disease-specific keywords related to bladder cancer, upper tract urothelial carcinoma, renal cell carcinoma, and prostate cancer. We primarily included clinical and translational studies, together with selected preclinical reports, that were directly relevant to the diagnostic performance, clinical utility, safety, limitations, and future applications of 5-ALA-based fluorescence guidance in urologic oncology. Because this article was designed as a narrative review rather than a systematic review, study selection and data extraction were not performed using a formal predefined protocol; instead, studies were selected and synthesized according to their relevance to the scope and aims of the review.

## 2. Bladder Cancer

Within urologic oncology, 5-ALA-mediated photodynamic diagnosis (PDD) has been most extensively studied and clinically implemented in bladder cancer, where enhanced cystoscopic visualization can mitigate missed lesions and incomplete resection. Contemporary guidelines support the use of photodynamic or blue-light-enhanced cystoscopy as an adjunct in selected settings for NMIBC, reflecting its value for detecting small lesions and CIS [21,22]. Specifically, AUA/SUO guidance recommends offering blue-light cystoscopy at the time of TURBT, if available, to increase detection and decrease recurrence [22]. The EAU guideline similarly notes that photodynamic diagnosis improves detection of bladder cancer, especially CIS, while cautioning that false-positivity can be induced by inflammation or recent TURB and during the first three months after BCG instillation [23].

Importantly, regional practice varies with respect to both the photosensitizer used and the route of administration. Early clinical development in Europe focused on intravesical 5-ALA, which improved visualization of dysplasia, CIS, and early papillary tumors and motivated subsequent outcome-focused trials [24,25]. In contrast, Japan has advanced oral 5-ALA regimens for PDD-guided TURBT, supported by investigator-initiated randomized studies and multicenter phase III evaluation, and this approach has become a distinctive feature of Japanese clinical practice [12,13,14,15]. Outside Japan, the most widely adopted approach for blue-light cystoscopy is intravesical hexaminolevulinate (HAL), which has been evaluated in large international studies for improved detection and, in some trials, reduced recurrence [26,27,28,29,30]. More recently, flexible blue-light cystoscopy has also been evaluated for NMIBC surveillance, and a meta-analysis suggested only a modest incremental yield for detecting recurrence (including CIS) compared with flexible white-light cystoscopy, at the cost of increased false-positive findings [31,32,33].

In the following section, because the primary focus of this review is 5-ALA, we first summarize the diagnostic performance of 5-ALA-based PDD in NMIBC, distinguishing oral and intravesical administration where relevant, and then contextualize these findings against HAL-based blue-light cystoscopy and other enhanced imaging modalities.

### 2.1. Diagnostic Performance (CIS, Post-BCG, False Positives)

Key clinical studies in NMIBC are summarized in Table 1, including differences in photosensitizers, administration routes, study designs, and endpoints (diagnostic accuracy, recurrence, and economic outcomes). Because the primary focus of this review is 5-ALA, we first summarize the evidence for 5-ALA-based PDD, distinguishing oral and intravesical administration where relevant, before comparing these data with HAL-based blue-light cystoscopy. Across multiple studies, 5-ALA-based PDD has improved detection of urothelial lesions that may be inconspicuous under white-light cystoscopy, particularly CIS and small or multifocal tumors [12,13,14,15,24].

Among current 5-ALA-based approaches, the best-characterized contemporary data come from oral administration in Japan. Subsequent Japanese trials of oral 5-ALA confirmed improved lesion detection and established dosing and timing parameters that remain central to current practice [13,14,15]. In a Japanese multicenter phase III study, lesion-based sensitivity increased from 54.1% with WL to 79.6% with oral 5-ALA PDD, whereas specificity decreased from 95.5% to 80.6% (corresponding false-positive rate, defined as 1 minus specificity, 4.5% vs. 19.4%) [14]. These data support oral 5-ALA as the most developed contemporary 5-ALA platform in NMIBC, while underscoring the need for careful interpretation of fluorescence-positive areas.

Intravesical 5-ALA represents the earlier developmental route and was used in foundational European studies demonstrating improved visualization of dysplasia, CIS, and early papillary tumors [24,25]. In a head-to-head comparison of oral versus intravesical 5-ALA, both approaches supported PDD-guided TURBT, while also illustrating practical differences in workflow, timing, and fluorescence intensity that remain relevant when interpreting route-specific performance in NMIBC [12]. Thus, oral and intravesical 5-ALA should be viewed as related but operationally distinct implementations of the same fluorophore platform.

When diagnostic accuracy is described using conventional metrics, the most reproducible trend is improved sensitivity with PDD relative to WL, while specificity and positive predictive value can be lower, particularly in the presence of inflammatory changes [13,14,15,34,35,36]. This trade-off is clinically relevant because it increases the likelihood of additional biopsies and may influence interpretation during mapping or resection. Reactive and treatment-related changes account for a substantial proportion of false-positive PDD findings, and both specificity and positive predictive value are lower among patients with prior BCG exposure than among BCG-naive patients [34,35,36]. Preoperative pyuria has also been associated with lower specificity and higher false-positive rates, suggesting that simple urine findings may help anticipate reduced diagnostic specificity in inflammatory settings [37]. Beyond endoscopic enhanced imaging, 5-ALA-induced fluorescent urine cytology has also been explored as an adjunct diagnostic application in bladder cancer. Preliminary studies have suggested higher sensitivity than conventional urine cytology while maintaining high specificity, with potential utility even in low-grade urothelial carcinoma, although this approach remains outside the main scope of fluorescence-guided cystoscopic visualization addressed in this review [38,39].

Whether improved detection translates into improved oncologic outcomes remains more heterogeneous across studies. In Japan, several real-world cohorts and comparative analyses using oral 5-ALA have suggested reductions in residual tumor and intravesical recurrence following PDD-assisted TURBT [16,17,18,40,41,42,43]. In the primary analysis of the prospective BRIGHT cohort in high-risk NMIBC, PDD-TURBT using oral 5-ALA was associated with a significantly lower residual tumor rate at second TURBT than white-light TURBT after propensity score matching (25.7% vs. 47.3%; odds ratio 0.39) [44]. In a subsequent BRIGHT analysis focused on intravesical recurrence after second TURBT, PDD-TURBT was associated with fewer recurrences within 1 year after propensity score matching (HR 0.44), with subgroup signals suggesting that the magnitude of benefit may depend on baseline tumor features and postoperative intravesical therapy [18]. These oral 5-ALA data suggest that therapeutic benefit may be achievable in carefully implemented settings, although most studies remain nonrandomized, and treatment heterogeneity should be considered when interpreting effect sizes.

As a comparative platform, intravesical HAL-based blue-light cystoscopy has accumulated the largest international trial and registry experience. Phase III and multicenter studies have shown improved detection of CIS and papillary tumors compared with white light, and some trials have suggested reductions in early recurrence or longer time to recurrence [26,27,28,29,30]. HAL offers the practical advantage of broad international validation and avoids systemic oral 5-ALA exposure, but it requires intravesical instillation and dwell time, and false-positive biopsies remain an issue in inflamed or recently treated bladders [23,26,27]. In contrast, oral 5-ALA provides a distinct systemic approach with growing real-world outcome data, particularly from Japan, but systemic adverse events such as hypotension and photosensitivity are more relevant [14,15,45].

However, pragmatic long-term data for HAL have been less uniformly positive. In the United Kingdom PHOTO trial, HAL-based PDD did not reduce recurrence at 3 years and was not cost-effective compared with WL TURBT [46,47]. Other cost and budget-impact analyses suggest that the incremental costs of HAL-based blue-light cystoscopy are highly context dependent [48,49,50]. Direct superiority claims between oral 5-ALA and HAL are therefore inappropriate because the fluorophore, route of administration, workflow, and post-treatment context differ substantially across studies. A direct comparison with narrow-band imaging (NBI) also suggested that fluorescence-based contrast may be particularly helpful for detecting flat lesions such as CIS that may be missed under non-fluorescence-enhanced imaging [51,52]. Consequently, prospective multicenter studies that harmonize imaging protocols, define post-treatment strata, and link fluorescence guidance to residual disease, recurrence patterns, safety, and patient-centered outcomes remain an important next step [12,21,22,46].

**Table 1:** Clinical evidence overview for 5-ALA (oral/intravesical) and HAL (intravesical) photodynamic diagnosis in NMIBC/CIS.

Study (Ref.)	Region	Photosensitizer/Route	Setting	Design	N	Key Endpoints	Key Take-Home	Main Pitfalls
Inoue et al., 2012 [12]	Japan	5-ALA (oral vs. intravesical)	TURBT (suspected NMIBC/CIS)	Comparative cohort	210 cases (75 intravesical; 135 oral)	Sensitivity/specificity (biopsy-level): 93.4%/58.9% (PDD) vs. 44.7%/94.1% (WL); Flat lesions detected only by PDD: 72.1% (oral 74.3% vs. intravesical 68.6%).	Oral 5-ALA enabled PDD and supported adoption in Japan	Lower specificity; inflammation/previous treatment may increase false positives
Inoue et al., 2015 [13]	Japan	5-ALA oral (dose evaluation)	TURBT (NMIBC)	Randomized, double-blind (dose)	62 patients	Sensitivity/specificity (20 mg/kg): 75.8%/68.2% (PDD) vs. 47.6%/95.9% (WL); PDD-only detection 29.8% vs. 1.6%; SAEs 4/62 (none 5-ALA-related)	Supported 20 mg/kg dosing with favorable detection	Low specificity with PDD (20 mg/kg): Spec 68.2% vs. 95.9% (WL) (FPR 31.8% vs. 4.1%); PPV 60.3% vs. 88.1%.
Nakai et al., 2018 [14]	Japan	5-ALA oral 20 mg/kg	TURBT (NMIBC)	Prospective multicenter phase III	61 patients (511 specimens)	Sensitivity 79.6% (PDD) vs. 54.1% (WL); Specificity 80.6% (PDD) vs. 95.5% (WL; lesion); FPR 19.4% (PDD) vs. 4.5% (WL)	High sensitivity for NMIBC including flat lesions	False positives in inflammatory areas (FPR 19.4% in PDD); systemic AEs (e.g., hypotension)
Yamamoto et al., 2020 [15]	Japan	5-ALA oral 20 mg/kg	PDD-TURBT (real-world)	Single-center real-world cohort	76 patients	Diagnostic sensitivity/specificity: 90.1%/61.2% (PDD) vs. 65.4%/88.9% (WL); Mean no. tumors per patient: 1.92 vs. 1.39.	Feasibility of oral 5-ALA-PDD in routine practice	Operator/workflow variability; lower specificity with PDD (61.2% vs. 88.9%; FPR 38.8% vs. 11.1%).
Hagimoto et al., 2021 [51]	Japan	5-ALA oral 20 mg/kg	TURBT (WL vs. PDD vs. NBI)	Comparative (lesion-based)	114 patients (282 lesions)	Sensitivity/specificity: 89.6%/22.5% (PDD) vs. 76.2%/46.3% (NBI); CIS sensitivity: 94.6% vs. 54.1%.	PDD improved sensitivity vs. NBI, particularly for CIS	Very low specificity for PDD (22.5%; FPR 77.5%); inflammation-related false positives.
Fukuhara et al., 2022 [16]	Japan	5-ALA oral	TURBT (recurrence outcome)	Retrospective comparative cohort	PDD 105 vs. WL 88	Intravesical recurrence: 20.0% (PDD, 21/105) vs. 33.0% (WL, 29/88); Recurrence frequency: 5.82 vs. 12.80 per 10,000 days.	Real-world data suggested reduced bladder recurrence after PDD-TURBT	Impact of resection of false positives; retrospective design.
Taoka et al., 2022 [17]	Japan	5-ALA oral	Initial TURBT and second TUR	Retrospective comparative cohort	PDD 115 vs. WL 346	Residual tumor: 10.3% (PDD, 11/107) vs. 33.8% (WL, 24/71); Intravesical recurrence: 9.6% vs. 41.9%; RFS HR 2.01 (WL vs. PDD).	Lower residual disease at second TUR and improved RFS with PDD	Nonrandomized; treatment heterogeneity (e.g., intravesical therapy schedules).
Kobayashi et al., 2022 (BRIGHT primary analysis) [44]	Japan	5-ALA oral	High-risk NMIBC (second TUR; residual tumor endpoint)	Prospective registry vs. retrospective; PSM	PDD 177 vs. WL 306 (matched 167/167)	Residual tumor at 2nd TURBT: 25.7% (PDD) vs. 47.3% (WL) (OR 0.39)	Residual tumor at 2nd TURBT: 25.7% vs. 47.3% (PDD vs. WL); risk model proposed for selective omission of 2nd TURBT.	Nonrandomized/historical control; confounding and practice heterogeneity
Kawai et al., 2024 (BRIGHT) [18]	Japan	5-ALA oral	High-risk NMIBC (after second TUR)	Prospective registry vs. retrospective; PSM	PDD 177 vs. WL 306 (matched 146/146)	1-year intravesical recurrence: HR 0.44 (PSM)	PDD-TURBT associated with lower short-term recurrence in high-risk NMIBC	Nonrandomized; benefit may vary by BCG use/tumor features
Fradet et al., 2007 [26]	Europe/N. America	HAL (intravesical)	Cystoscopy/TURBT (CIS detection)	Phase III multicenter	58 patients	CIS lesions detected: 92% (104/113) (HAL) vs. 68% (77/113) (WL); In CIS patients, ≥1 CIS lesion found only by HAL in 41.5% vs. WL-only 15.1%.	Improved detection of CIS compared with WL	False-positive biopsy rate: 39% (HAL) vs. 31% (WL); inflammation/instillation effects; learning curve.
Grossman et al., 2007 [27]	N. America/Europe	HAL (intravesical)	Cystoscopy/TURBT (papillary lesions)	Phase III multicenter	196 evaluable	Additional tumor detection (Ta): ≥1 more tumor with HAL in 29% (31/108); Ta detection rate 95% vs. 83%; T1 detection rate 95% vs. 86%; additional T1 detected in 15% (3/20).	HAL fluorescence found additional Ta/T1 lesions missed by WL	False detection rate (biopsy-level): 39% (HAL) vs. 31% (WL). Center/operator variability (training cases excluded); equipment/training required.
Stenzl et al., 2010 [28]	Multinational	HAL (intravesical)	TURBT (NMIBC)	RCT (HAL-PDD vs. WL)	814 patients	Recurrence at 9 months: 47% vs. 56% (HAL vs. WL)	HAL-PDD reduced residual tumor and early recurrence	Effect size modest (RRR 16%); Outcomes depend on TURBT quality and adjuvant therapy
Grossman et al., 2012 [29]	Multinational	HAL (intravesical)	TURBT (NMIBC)	Long-term follow-up	551 participants	Median time to recurrence: 16.4 vs. 9.4 months (HAL vs. WL); Tumor-free at ~53–55 mo: 38.0% vs. 31.8%.	Sustained reduction in recurrence and longer time-to-recurrence	Intravesical therapy rates similar (45% vs. 46%); follow-up extension design.
Gallagher et al., 2017 [30]	UK	HAL (intravesical)	New NMIBC TURBT	Prospective controlled study	345 analyzed (808 recruited)	3-year recurrence: 39.0% vs. 53.3% (OR 0.56); matched 24.6% vs. 50.0% (OR 0.33); high-risk 52.1% vs. 80%.	Lower recurrence at 3 years with “good-quality” PDD-TURBT vs. WL	Nonrandomized pre/post design; potential confounding despite “good quality” criteria.
Lotan et al., 2021 [31]	USA	HAL (intravesical)	Flexible cystoscopy (surveillance)	Prospective phase III	190 patients (322 procedures)	WLC−/BLFC+: 8% (biopsied cancers 87%); WLC+/BLFC+: 25.8% (additional tumors 33%).	Blue-light flexible cystoscopy improved detection during surveillance	Benign findings: 3 cases in WLC−/BLFC+ (<1% cohort); benign biopsies in 25% (office) and 12% (operating room) of WLC+/BLFC+ cases.
Pohar et al., 2022 [32]	USA	HAL (intravesical)	Surveillance and OR TURBT (repeat HAL)	Prospective phase III (safety)	310 total; repeat dose subset 103	HAL-related AEs: 2.2% (surveillance) vs. 3.4% (post-TURBT); repeat HAL dosing 33.2% (103 patients).	Repeat HAL administration appeared safe	Short-term AE capture; HAL-related events were rare.
Motlagh et al., 2024 [33]	Mixed	HAL/BLC (intravesical)	Surveillance (systematic review/meta-analysis)	Meta-analysis	10 studies; 1634 patients	Surveillance BLC meta-analysis: additional detection RD 0.045 (4.5%); recurrence RR 0.81 (*p* = 0.002); false positives RR 0.89 (not significant).	BLC showed incremental lesion detection in surveillance settings	Heterogeneous study designs and endpoints; limited high-quality comparative data.
Heer et al., 2022 (PHOTO) [46]	UK	PDD (routine practice)	TURBT (suspected NMIBC)	Pragmatic multicenter RCT	538 enrolled	PHOTO trial: time to recurrence HR 0.94; 3-year RFS 57.8% vs. 61.6% (PDD vs. WL); cost +£876, QALY −0.007.	No clear reduction in recurrence vs. WL in pragmatic implementation	No reduction in recurrence at 3 years; not cost-effective vs. WL.

Abbreviations: 5-ALA, 5-aminolevulinic acid; HAL, hexaminolevulinate; PDD, photodynamic diagnosis; WL, white light; WLC, white-light cystoscopy; BLC, blue light cystoscopy; BLFC, blue light flexible cystoscopy; NBI, narrow-band imaging; TURBT, transurethral resection of bladder tumor; NMIBC, non-muscle-invasive bladder cancer; CIS, carcinoma in situ; BCG, bacillus Calmette–Guérin; RFS, recurrence-free survival; PSM, propensity score matching; FPR, false-positive rate; PPV, positive predictive value; AE, adverse event; SAE, serious adverse event; OR, odds ratio; HR, hazard ratio; RR, risk ratio; RD, risk difference; RRR, relative risk reduction; QALY, quality-adjusted life year; RCT, randomized controlled trial.

### 2.2. Adverse Events: Hypotension, Hepatic Dysfunction, and Photosensitivity

Oral 5-aminolevulinic acid (5-ALA) for photodynamic diagnosis (PDD) in transurethral resection of bladder tumor (TURBT) is generally well tolerated in prospective clinical trials and real-world practice; however, several adverse events remain clinically relevant because they can influence anesthetic management, postoperative observation, and discharge planning [13,14,15,53]. In a multicenter phase III study of oral 5-ALA-mediated PDD, no grade 4–5 adverse events were observed, while hypotension and urticaria were the main severe events for which a causal relationship with 5-ALA could not be excluded [14]. Post-marketing and institutional series further highlight hypotension and transient elevations in liver enzymes as recurrent safety signals, emphasizing the importance of perioperative risk stratification rather than blanket avoidance of 5-ALA [15].

Hypotension is the most frequently discussed perioperative adverse event with oral 5-ALA-PDD-TURBT, but its reported incidence varies substantially across studies, largely reflecting differences in definitions (e.g., minimum systolic blood pressure (SBP) ≤ 80 mmHg vs. broader “any-grade” hypotension) and anesthetic practice [16,45,54,55,56,57,58]. In a multicenter retrospective cohort (*n* = 245), 63.7% of patients experienced any-grade hypotension during ALA-PDD-TURBT, and multivariable analysis identified a history of hypertension and general anesthesia as independent risk factors, whereas calcium channel blocker use was associated with a lower risk of hypotension [59]. In another institutional analysis comparing ALA-PDD-TURBT with conventional TURBT, general anesthesia and regular use of renin–angiotensin system (RAS) inhibitors were associated with SBP drops below 80 mmHg, and the authors suggested considering perioperative withdrawal of RAS inhibitors and close blood pressure monitoring [54]. Complementing these data, a risk–benefit analysis focused on patient selection reported intraoperative hypotension (SBP < 80 mmHg) in 35.6% of PDD cases and identified age ≥ 75 years and general anesthesia as independent predictors [55]. In elderly patients (over 70 years), comparative data also suggested that intraoperative hypotension (SBP ≤ 80 mmHg) occurred more frequently in the PDD group than in the white-light TURBT group, supporting heightened caution when introducing 5-ALA in older populations [57]. Conversely, in a more recent single-center study restricted to spinal anesthesia cases, hypotension (SBP ≤ 80 mmHg) remained common (58.7%), but no strong patient-related predictors were identified; nausea and vomiting were associated with phenylephrine use [58]. Taken together, these reports indicate that risk factors for hypotension are not fully consistent across cohorts, but anesthetic modality, baseline cardiovascular status (including hypertension), and perioperative antihypertensive management (notably RAS inhibitors), and renal function impairment (reduced eGFR) repeatedly emerge as practical considerations for multidisciplinary planning [16,54,55,58].

Beyond baseline characteristics and medication history, peri-induction blood pressure dynamics may provide an additional practical signal. In a retrospective cohort comparing ALA-PDD-TURBT with conventional TURBT, intraoperative hypotension requiring vasopressors was more frequent in the PDD group (43% vs. 17%). Among patients receiving ALA-PDD-TURBT, a smaller increase in mean arterial pressure (MAP) from awakening to anesthesia induction (<+6.5 mmHg) independently predicted intraoperative hypotension, alongside advanced age and general anesthesia. Although this parameter cannot be used to preselect patients before 5-ALA intake, it may serve as a simple early-warning marker at induction to prompt closer hemodynamic monitoring and proactive management [60].

Timing optimization between oral 5-ALA intake and anesthesia/surgery is an emerging, low-burden mitigation strategy. Following a 2024 revision of the recommended administration window to allow intake 2–8 h preoperatively, a retrospective study comparing an interval <4 h versus ≥4 h from 5-ALA intake to anesthesia induction reported fewer moderate-to-severe hypotension events in the ≥4 h group (38% vs. 61%) and lower vasopressor requirements. Together with phase III data suggesting that extending the dosing-to-procedure interval does not materially compromise PDD diagnostic performance, deliberate scheduling to extend the intake-to-anesthesia interval may be particularly useful in patients with known risk factors (e.g., older age, renal dysfunction, and RAS inhibitor/ARB use) [61].

Reported adverse event profiles for oral 5-ALA-PDD-TURBT are summarized in Table 2, including definitions used, incidence ranges, severity, reported predictors, and practical mitigation strategies.

Transient hepatic dysfunction is another adverse event that warrants structured counseling and selective biochemical monitoring. A phase III trial report specifically highlighted transient liver toxicity after oral 5-ALA in bladder cancer patients undergoing PDD-TURBT [63], and later studies have attempted to identify clinical predictors and contextualize its severity. In a recent exploratory analysis of the prospective phase III SPP2C102 trial (*n* = 145), liver enzyme elevations occurred in 19.3% of patients, all grade 1–2, with systolic blood pressure ≥ 130 mmHg independently associated with liver enzyme elevations, suggesting that simple bedside parameters may guide closer postoperative laboratory follow-up [62]. In addition, an institutional risk factor analysis reported postoperative liver dysfunction in 51.9% of cases and identified RAS inhibitor use as a significant risk factor, although differences in definitions and timing of laboratory testing likely contribute to variability in incidence estimates across studies [56]. In an elderly cohort comparison, AST/ALT elevation was also significantly more frequent in the PDD group than in the white-light TURBT group, reinforcing the notion that age-stratified monitoring strategies may be appropriate when using oral 5-ALA in routine practice [57]. Overall, the available evidence suggests that hepatic enzyme elevations are usually mild and self-limited, yet sufficiently common to justify baseline assessment and targeted postoperative testing in higher-risk patients [57,62,63].

**Figure 2:**
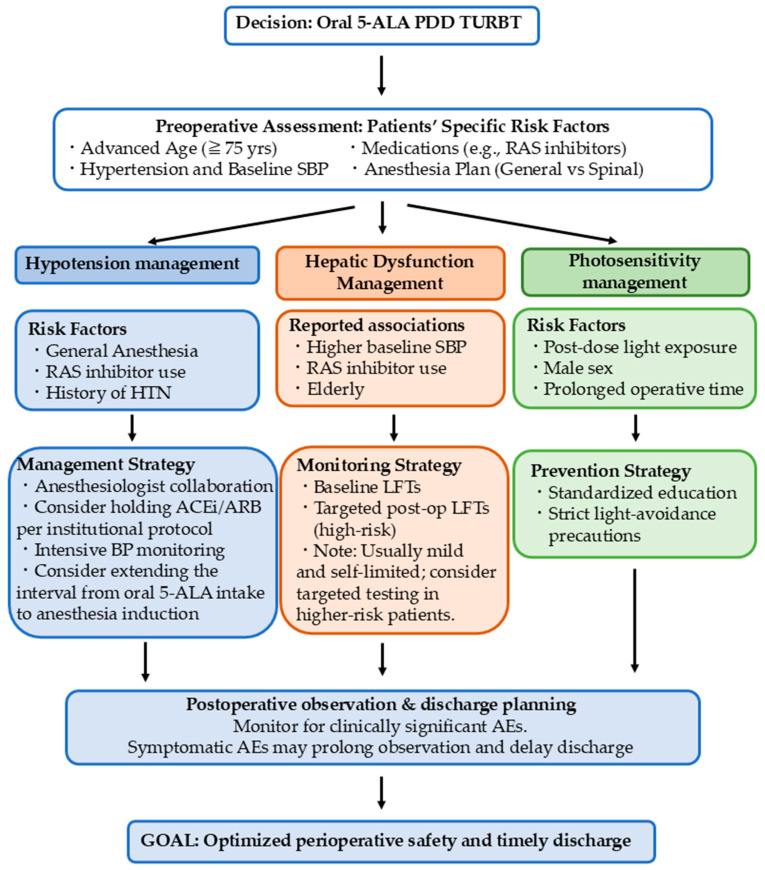
Proposed perioperative risk assessment and management pathway for adverse events associated with oral 5-ALA guided PDD-TURBT. The flowchart summarizes practical evaluation and mitigation strategies for the main clinically relevant adverse events reported with oral 5-ALA during TURBT, including hypotension, transient hepatic enzyme elevations, and photosensitivity. Key patient and perioperative factors (for example, advanced age, baseline blood pressure, antihypertensive regimens such as renin-angiotensin system inhibitors, and anesthesia plan) are incorporated to guide perioperative planning and postoperative monitoring. The overall goal is to optimize perioperative safety and facilitate timely discharge in the short-stay TURBT setting. Abbreviations: 5-ALA, 5-aminolevulinic acid; PDD, photodynamic diagnosis; TURBT, transurethral resection of bladder tumor; AE, adverse event; BP, blood pressure; SBP, systolic blood pressure; RAS, renin-angiotensin system; ACEi/ARB, angiotensin-converting enzyme inhibitor/angiotensin receptor blocker; LFTs, liver function tests.

Photosensitivity is a mechanistically plausible class effect of systemic 5-ALA administration and remains an important component of patient education. In an institutional cohort evaluating multiple adverse events, photosensitivity was observed in 31.7% of patients, with male sex and longer operative time reported as associated factors [56]. While the clinical spectrum is often mild with appropriate light-avoidance guidance, these data support incorporating standardized counseling and post-dose precautions into perioperative pathways, particularly for patients expected to have prolonged operative or hospital exposure [56]. In summary, because TURBT is typically designed for short-stay management, clinically significant hypotension or symptomatic adverse events may prolong postoperative observation and delay discharge. A practical perioperative risk assessment and management pathway for oral 5-ALA-PDD-TURBT is proposed in Figure 2. Therefore, urologists should weigh the benefits of improved tumor detection and potential reduction in recurrence against patient-specific risk factors (e.g., advanced age, hypertension, anesthetic plan, and antihypertensive regimen) and collaborate closely with anesthesiologists to optimize perioperative safety when considering oral 5-ALA [54,55,57,58,59].

## 3. Upper Tract Urothelial Carcinoma

Upper tract urothelial carcinoma (UTUC) arises from the same urothelial lineage as bladder cancer; therefore, fluorescence-based detection with 5-ALA is conceptually appealing, particularly for subtle lesions such as CIS. Current clinical pathways combine cross-sectional imaging, urinary cytology, and diagnostic ureterorenoscopy (URS) with biopsy; however, endoscopic recognition and mapping of flat or multifocal disease can remain challenging under conventional WL, which may contribute to underdiagnosis or incomplete endoscopic assessment [64].

Evidence for 5-ALA-mediated photodynamic diagnosis ureterorenoscopy (PDD-URS) remains limited but is consistently supportive of improved lesion conspicuity. In a systematic review assessing PDD in UTUC, seven studies (*n* = 194) were included; in the largest available cohort (*n* = 106), sensitivity was 95.8% for PDD-URS versus 53.5% for WL-URS, with similar specificity (96.6% vs. 95.2%; corresponding false-positive rate, 3.4% vs. 4.8%) [65]. In ureteroscopic settings—particularly in the ureter where visualization is often oblique—tangential artifacts and inflammatory changes can contribute to false-positive fluorescence, and changing the angle of inspection has been suggested to verify fluorescence before sampling [65]. In an early prospective audit, oral 5-ALA enabled PDD-URS to detect additional fluorescent lesions not evident under WL inspection, suggesting potential incremental value for targeted sampling in selected patients [66]. Subsequent prospective studies using oral 5-ALA reported higher sensitivity for UTUC detection with PDD-URS than with WL-URS, with particular utility for identifying CIS lesions under blue-light mode, although the cohorts were small (e.g., 10–20 patients in representative studies) [67,68]. A prospective pilot cohort study using a contemporary video platform further supported feasibility in routine diagnostic URS workflows [69].

A recent single-center analysis by Sano and colleagues compared diagnostic performance between suspected UTUC evaluated by PDD-URS and suspected bladder urothelial carcinoma evaluated by PDD-TURBT, reporting that per-biopsy diagnostic performance for PDD in the UTUC cohort was at least comparable to that observed in the bladder cohort, while emphasizing that UTUC sample sizes remain modest [70]. Beyond diagnosis, fluorescence guidance is also being explored for kidney-sparing endoscopic management, including PDD-guided ureteroscopic laser ablation in a phase 2 single-arm trial, but broader validation is still required [71].

From a practical standpoint, most UTUC fluorescence studies have used oral 5-ALA, which avoids the need for intraluminal drug instillation and dwell time within the renal pelvis or ureter, steps that can be technically demanding and less standardized across centers [66,67,68,69]. Systemic adverse events, particularly hypotension, have been evaluated in PDD-URS cohorts; however, given that safety considerations of oral 5-ALA are addressed in detail in Section 2.2, they are not repeated here [72]. Overall, current data suggest that oral 5-ALA-PDD-URS is a promising adjunct to WL-URS for selected UTUC scenarios, especially in suspected CIS, but the evidence base remains limited, and larger, prospective, multicenter studies with standardized interpretation criteria and clinically meaningful endpoints are needed [64,68,69,70,71].

## 4. Renal Cell Carcinoma

Partial nephrectomy has become a cornerstone in the management of small renal masses, aiming to achieve oncological control while preserving renal function. Contemporary guidelines emphasize nephron-sparing surgery whenever feasible, particularly for clinical T1 tumors, and minimally invasive approaches have expanded accordingly [73,74]. In parallel, the technical complexity of partial nephrectomy has increased as surgeons tackle more challenging tumor locations (e.g., endophytic or hilar lesions), for which accurate intraoperative identification of tumor borders and resection margins remains a critical determinant of success.

Multiple adjuncts have been explored to support intraoperative navigation during partial nephrectomy. Near-infrared fluorescence (NIRF) imaging with indocyanine green (ICG), for example, can facilitate real-time visualization of renal vasculature and perfusion, and delayed imaging may help differentiate tumor from surrounding parenchyma in some settings [75]. However, fluorescence patterns with ICG do not reliably predict malignancy or histology, and additional strategies, such as preoperative super-selective tumor marking, have been proposed for technically demanding endophytic masses [76,77]. These experiences highlight both the promise and limitations of fluorescence-based guidance in kidney surgery, and they provide a clinically relevant context for 5-ALA-mediated PDD.

### 4.1. Clinical Evidence of 5-ALA–PDD in RCC Surgery

Early work suggested that systemic 5-ALA could generate PpIX fluorescence sufficient to delineate RCC margins. Popken and colleagues demonstrated clear fluorescence in xenograft models and reported a small pilot clinical series in which orally administered 5-ALA enabled macroscopic visualization of tumor borders during kidney-preserving resection [78]. Subsequently, Hoda and Popken reported a prospective single-center experience of fluorescence-guided laparoscopic partial nephrectomy (LPN) in 77 patients, in which most RCC lesions exhibited red fluorescence under excitation light and the reported diagnostic performance (including high sensitivity and specificity) appeared favorable; importantly, PDD was also described as identifying cases with positive resection margins [19].

In contrast, the utility of 5-ALA–PDD during robot-assisted partial nephrectomy (RAPN) has been less convincing in contemporary series. Matsuoka and colleagues evaluated 5-ALA–PDD during RAPN and found that intraoperative fluorescence was observed in only one tumor before renal artery clamping, and that fluorescence could also be present in peritumoral normal tissues [20]. Ex vivo assessment further demonstrated that fluorescence patterns could be discordant between tumor and normal tissue, consistent with heterogeneous PpIX biology and limited margin visualization under current operative conditions [20]. Collectively, these data suggest that clinical performance may depend on surgical modality, optical environment, and tumor biology, and that results obtained in LPN may not directly translate to RAPN without optimization.

### 4.2. Biological and Optical Determinants of Variable Fluorescence

The inconsistent clinical signal in RCC likely reflects a convergence of biological and technical factors. From a biological standpoint, intracellular PpIX accumulation after 5-ALA administration is shaped by both bioconversion to heme and transporter-mediated efflux. Experimental work indicates that variability in ABCG2 activity can strongly influence extracellular PpIX levels, supporting the concept that active efflux may reduce detectable tumor fluorescence [10,11]. In addition, FECH (ferrochelatase), which catalyzes the conversion of PpIX to heme, may modulate net PpIX retention; however, available data in RCC are not uniform across models. A gene-expression study in renal cancer cell lines suggested differential expression of enzymes involved in porphyrin metabolism, including FECH, compared with non-tumor renal cells [79], whereas kinetic analyses in RCC cell lines reported higher FECH Vmax than in a non-tumor HK-2 cell line, implying efficient PpIX-to-heme conversion as a potential mechanism of reduced fluorescence [11]. These mixed findings support a cautious interpretation: in RCC, PpIX accumulation appears heterogeneous, and the tumor-to-normal fluorescence ratio may be limited by a combination of ABCG2-mediated efflux and FECH-dependent bioconversion, with the dominant mechanism potentially varying by tumor subtype, cellular context, and timing [10,11,79].

Technical and optical considerations are similarly important. Blue-light excitation and the need to distinguish a relatively weak red fluorescence signal in a bright operative field create practical challenges, particularly during RAPN. The observation that fluorescence could be seen only under specific conditions (e.g., with reduced background illumination) and that normal parenchyma may fluoresce in some cases underscores the need for standardized imaging protocols and possibly quantitative fluorescence detection rather than purely visual assessment [20]. Tumor location may further matter: endophytic tumors are intrinsically difficult to localize and dissect, and any optical method that depends on surface excitation and emission is vulnerable to depth-related signal loss, which may contribute to false-negative fluorescence in deeply embedded lesions [20,77].

Overall, current evidence indicates that 5-ALA-based fluorescence guidance in RCC is biologically and technically constrained by heterogeneous PpIX accumulation, potential background signal from normal renal parenchyma, and limited depth penetration in endophytic tumors, particularly in the optical environment of robot-assisted surgery. These factors likely contribute to the discrepancy between favorable laparoscopic reports and less consistent findings in contemporary robotic series. Accordingly, 5-ALA-guided margin visualization in RCC should be considered investigational at present. Strategies to overcome these limitations, including pharmacologic signal amplification (e.g., targeting porphyrin efflux and heme conversion pathways), improvements in camera sensitivity and quantitative fluorescence readouts, and standardized multicenter study designs, are discussed in detail in Section 6.

## 5. Prostate Cancer

In prostate cancer surgery, positive surgical margins (PSMs) remain a clinically relevant concern because they can compromise oncologic control while surgeons simultaneously strive to preserve continence and erectile function. Intraoperative tools capable of identifying residual cancer on the prostatic surface could, in principle, support immediate additional resection in selected areas. Within this context, 5-ALA-mediated PDD has been explored as an intraoperative fluorescence approach to visualize PpIX at suspected margin sites during radical prostatectomy [80,81,82].

The earliest clinical experience by Zaak and colleagues demonstrated selective PpIX accumulation in malignant prostatic epithelium and suggested that fluorescence assessment of surgical margins is technically feasible during radical prostatectomy [80]. Subsequently, a multicenter, prospective, phase 2 diagnostic trial evaluated 5-ALA–PDD for intraoperative identification of PSMs during open and endoscopic extraperitoneal radical prostatectomy, supporting its feasibility and providing early estimates of diagnostic accuracy [81]. Additional series in endoscopic extraperitoneal and open approaches further described operative workflows and margin-directed assessment using dedicated PDD endoscopic systems [82,83]. A small report also demonstrated technical integration of 5-ALA–PDD into robot-assisted laparoscopic radical prostatectomy (RALP) using a dual-display setup, although the study included only a limited number of cases and did not establish reliability for detecting true-positive margins [84].

Despite these encouraging feasibility signals, clinically important limitations constrain 5-ALA–PDD in prostatectomy. In particular, Fukuhara and colleagues highlighted that short linear PSMs can substantially reduce detectability, and heat generated by energy devices may quench or destroy intracellular PpIX, thereby masking fluorescence [85]. The same study emphasized a fundamental optical constraint: blue-light excitation has shallow tissue penetration, limiting fluorescence assessment largely to surface-exposed tumors; consequently, extraprostatic extension covered by capsule or periprostatic fat may not generate a visible signal on the specimen surface [85]. These limitations are conceptually analogous to those seen in other organs, where fluorescence contrast and optical access (including disease depth) largely determine clinical utility.

Taken together, current evidence supports the feasibility of 5-ALA–PDD for intraoperative assessment during radical prostatectomy, but available clinical data remain limited and heterogeneous, and there is no established standard-of-care role at present. Recent reviews of intraoperative PSM assessment technologies underscore that multiple approaches are under active development, including targeted tracers and advanced imaging platforms, but robust demonstrations of outcome benefit and scalable implementation are still evolving [86]. In this review, broader cross-disease strategies to improve fluorescence contrast, quantification, and clinical trial design are discussed in Section 6.

## 6. Comparative Context and Future Directions

### 6.1. Comparative Context with Other Optical Imaging Modalities

Before considering future directions, it is useful to position 5-ALA-based fluorescence guidance alongside other adjunct optical platforms in urologic oncology. Such comparisons should be interpreted cautiously because these technologies serve partly different clinical purposes. 5-ALA-PDD and NBI are primarily wide-field lesion detection tools, probe-based confocal laser endomicroscopy (pCLE) provides microscopic characterization of suspicious mucosal lesions, and ICG near-infrared fluorescence mainly supports vascular and perfusion assessment during renal surgery rather than tumor-selective detection [51,75,76,87].

In this context, 5-ALA currently retains its clearest advantage for fluorescence-guided identification of flat urothelial lesions such as CIS and for lesion mapping during resection, whereas NBI offers drug-free contrast enhancement, pCLE offers real-time optical biopsy, and ICG is most useful as an anatomic and perfusion adjunct. Table 3 summarizes the distinct translational niches, representative performance signals, and major caveats of each approach [14,26,28,51,76,87].

### 6.2. Future Directions in Urothelial Malignancies

The broader clinical application of 5-ALA-guided endoscopic surgery in urothelial malignancies is currently limited by several unresolved challenges. First, improved lesion detection does not uniformly translate into durable oncologic benefit across study designs and practice settings. Second, cross-study interpretation remains difficult because outcomes are influenced by heterogeneity in patient risk, adjuvant treatment, surgical quality, fluorophore choice, and workflow, including differences between oral 5-ALA and intravesical HAL. Third, systemic adverse events associated with oral administration, particularly hypotension, create implementation barriers that require structured perioperative risk management. Finally, in upper tract urothelial carcinoma, the available evidence remains promising but limited, with small cohorts and insufficient multicenter standardization of lesion mapping and clinically relevant endpoints.

To overcome these barriers, future studies should move beyond lesion-level detection alone and adopt harmonized, clinically meaningful endpoints such as residual tumor burden, recurrence, progression, patient-reported outcomes, and cost-effectiveness. Prospective multicenter trials should standardize route-specific workflows and explicitly account for perioperative safety, particularly for oral 5-ALA. In parallel, reproducibility may be improved by calibration-aware imaging, standardized reporting of optical parameters, and quantitative or device-aware fluorescence assessment. Emerging computational approaches, including artifact correction, digital contrast enhancement, and artificial intelligence-assisted cystoscopy, may further reduce operator dependence. In addition, optimizing administration-to-imaging timing and establishing standardized lesion mapping frameworks will be important for extending 5-ALA-guided imaging more reliably to UTUC.

Current evidence for 5-ALA-based PDD in urologic oncology is most mature in urothelial cancers, particularly NMIBC. Prospective and real-world studies of oral 5-ALA fluorescence cystoscopy consistently demonstrate improved tumor visualization compared with white-light cystoscopy, providing a basis for broad clinical adoption in Japan [14,15]. In parallel, intravesical blue-light cystoscopy using HAL has accumulated robust trial-level evidence for enhanced detection of carcinoma in situ and papillary disease, and several studies have suggested a reduction in early recurrence after TURBT [26,27,28,29,88]. Collectively, these data indicate that fluorescence-guided resection is a clinically credible platform for improving surgical completeness in urothelial malignancies.

However, translating improved detection into durable oncologic benefit remains an unresolved, methodologically sensitive question. A large pragmatic randomized trial in the United Kingdom reported that PDD-guided TURBT did not reduce 3-year recurrence compared with white-light TURBT and was not cost-effective, underscoring that “improved visualization” does not automatically guarantee better long-term outcomes in routine practice [46]. Differences in surgical quality, use of second resection, adjuvant intravesical therapy, risk composition, and institutional experience may all modulate whether PDD produces a measurable recurrence benefit, and future trials should prospectively standardize these variables while using clinically meaningful endpoints beyond lesion-level detection [46].

For oral 5-ALA-guided TURBT, multiple contemporary Japanese real-world datasets have reported lower residual tumor burden at second resection and/or improved intravesical recurrence-free survival compared with white-light TURBT, suggesting that therapeutic benefit is achievable in carefully implemented settings [16,17,18]. Nonetheless, cross-study comparisons are complicated by heterogeneity in patient risk, perioperative workflows, and the fluorophore/route used (oral 5-ALA versus intravesical HAL), making direct inference about “which approach is superior” inappropriate without harmonized protocols. This gap supports the need for international, prospective multicenter studies designed to (i) compare standardized PDD-TURBT workflows across regions and (ii) test whether fluorophore choice and administration route influence not only detection metrics but also recurrence, progression, patient-reported outcomes, and cost [26,27,28,29,46,88]. In addition, workflow optimization may further facilitate real-world implementation; for example, extending the permitted interval between oral 5-ALA administration and PDD evaluation has been investigated to improve logistical feasibility without sacrificing diagnostic performance [41]. Because systemic adverse events (notably hypotension and transient liver enzyme elevations) are more relevant to oral administration, future multicenter efforts should embed pragmatic risk-mitigation algorithms and explicitly report safety alongside effectiveness.

Beyond workflow refinement and trial harmonization, the next step for fluorescence-guided endoscopic surgery is to improve reproducibility by moving from purely visual interpretation toward quantitative, device-aware assessment. Notably, the apparent PpIX fluorescence intensity observed on screen can differ across cystoscopic imaging systems, supporting the need for calibration phantoms and standardized reporting of optical settings and working distance in multicenter studies [89]. In parallel, computational methods are rapidly maturing: artifact correction has been shown to enhance the image quality of blue-light cystoscopy [90], and digital staining of standard white-light cystoscopy videos has been proposed to generate blue-light-like contrast without exogenous agents, potentially lowering barriers to adoption while improving cross-platform consistency [91]. Artificial intelligence (AI)-based cystoscopy has also demonstrated strong diagnostic performance in large multicenter datasets and could be adapted to fluorescence cystoscopy as real-time decision support by integrating morphological and fluorescence cues, thereby reducing inter-operator variability [92,93,94]. Finally, emerging chronobiology data suggest that intracellular PpIX accumulation after 5-ALA exposure may exhibit circadian patterns, raising the possibility that optimizing the timing of PDD-TURBT could improve tumor-to-background contrast without increasing drug dose [95].

Upper tract urothelial carcinoma (UTUC) represents another promising but still evidence-limited frontier. Because UTUC arises from the same urothelial lineage, fluorescence-guided detection during ureterorenoscopy is biologically plausible, and prospective and retrospective studies of oral 5-ALA-PDD have reported improved diagnostic yield compared with white light [68,70]. The next major step is expansion to sufficiently powered multicenter cohorts with standardized lesion mapping and clinically relevant endpoints in kidney-sparing management (e.g., detection of flat lesions, completeness of endoscopic ablation, and subsequent recurrence patterns) [68,70].

### 6.3. Future Directions: Toward More Reliable RCC Guidance

Current limitations of 5-ALA-guided surgery in RCC should first be clearly acknowledged. Clinical studies have shown that intraoperative fluorescence can be inconsistent, tumor-to-background contrast may be inadequate, and fluorescence may overlap with normal renal parenchyma, particularly in robot-assisted settings. These limitations likely reflect both biological heterogeneity in PpIX handling and technical factors such as illumination geometry, working distance, and camera sensitivity. Accordingly, broader clinical application in RCC remains constrained not only by insufficient contrast, but also by the lack of standardized protocols and clinically meaningful validation endpoints.

Given the mixed clinical outcomes to date, future progress in 5-ALA-guided renal cell carcinoma (RCC) surgery will likely require both biological and technological optimization. On the biological side, ABCG2 inhibition is an attractive strategy because it targets a defined protoporphyrin IX (PpIX)-reducing mechanism; in RCC cell lines, ABCG2 inhibitors have been shown to enhance 5-ALA-induced PpIX fluorescence, supporting the feasibility of pharmacologic signal amplification [10]. Complementary approaches such as iron chelation (e.g., deferoxamine) have also been explored to increase 5-ALA-mediated fluorescence in RCC models [11]. Importantly, enhancement strategies must be evaluated not only for absolute fluorescence intensity but also for improvement in tumor-to-normal contrast, because any approach that increases PpIX in both tumor and normal renal tissue may fail to improve intraoperative discrimination [11,20]. Therefore, biomarker-informed selection (e.g., tumor ABCG2 activity or other determinants of PpIX handling) may ultimately be necessary to identify patients most likely to benefit from 5-ALA-PDD in RCC [10,11].

On the technological side, improvements in camera sensitivity, spectral filtering, and objective quantification could mitigate limitations of subjective fluorescence interpretation, particularly in robot-assisted partial nephrectomy, where illumination geometry and working distance differ from laparoscopy [20]. More broadly, the rapid evolution of optical guidance during partial nephrectomy, including near-infrared fluorescence approaches (e.g., indocyanine green for perfusion and anatomy), suggests that integrated, multimodal guidance rather than reliance on a single fluorophore may be the most realistic path forward for complex renal tumors [75,76,77]. In this context, 5-ALA should be viewed as a platform technology that may become clinically valuable in RCC once determinants of poor contrast are better controlled and prospective multicenter evaluations define standardized protocols and clinically meaningful endpoints [10,11,20].

## 7. Conclusions

In conclusion, 5-ALA-based fluorescence guidance currently has its clearest established clinical role in bladder cancer, particularly for photodynamic diagnosis during TURBT, where it improves visualization of carcinoma in situ and additional urothelial lesions beyond white-light cystoscopy [12,14,15]. However, broader clinical implementation remains limited by false-positive findings in inflammatory or post-treatment settings, heterogeneity in long-term oncologic benefit across study designs and workflows, and systemic adverse events associated with oral administration [45,46,54,58]. Outside bladder cancer, applications in upper tract urothelial carcinoma, renal cell carcinoma, and prostate cancer remain investigational because current studies are small, technically variable, and insufficient to define standardized protocols or routine clinical roles [20,68,70,85]. Key priorities for future investigation include harmonized multicenter trials, route- and platform-specific safety and imaging protocols, quantitative and device-aware fluorescence assessment, and clinically meaningful endpoints linking fluorescence guidance to residual disease, recurrence, margin status, and patient-centered outcomes [20,46,89].

## Figures and Tables

**Table 2:** Summary of reported adverse events associated with oral 5-ALA–PDD-assisted TURBT.

AE Type	Study (Ref.)	Definition Used	Incidence	Severity/Course	Reported Predictors	Practical Mitigation
Hypotension	Nohara et al., 2019 [54]	Vasopressor use during anesthesia/surgery (risk analysis performed using SBP < 80 mmHg)	Vasopressor use: PDD 50/109 (45.9%) vs. conventional TURBT 18/83 (21.7%)	Lower SBP and higher vasopressor use vs. historical controls	General anesthesia; regular use of renin-angiotensin system inhibitors	Consider withholding RAS inhibitors; close intraoperative BP monitoring
Hypotension	Fukuhara et al., 2021 [59]	Minimum SBP ≤ 80 mmHg (and graded hypotension)	Any grade hypotension: 156/245 (63.7%)	Typically transient; decision-tree risk stratification proposed	Hypertension; general anesthesia; calcium antagonists associated with lower risk	Pre-op risk assessment; anesthesia planning; medication review; enhanced monitoring
Hypotension	Nohara et al., 2024 [45]	Severity classification (institutional)	Moderate-to-severe: PDD 123/274 (44.9%) vs. WL 22/133 (16.5%); Severe: PDD 9/274 (3.3%) vs. WL 1/133 (0.8%)	Focus on clinically troublesome severe hypotension subset	Renal function impairment; other 5-ALA adverse effects; anesthesia type may affect severity	Identify high-risk patients; tailor anesthesia/monitoring; manage other AEs proactively
Hypotension	Kobayashi et al., 2023 [55]	SBP <80 mmHg	79/222 (35.6%) in PDD group	Intraoperative; weighed against recurrence reduction	Age ≥ 75 years; general anesthesia	Cautious patient selection in elderly; anesthesiology collaboration; enhanced monitoring
Hypotension	Ishikawa et al., 2024 [60]	Hypotension requiring vasopressors during TURBT	80/184 (43%) (PDD-TURBT) vs. 50/303 (17%) (conv-TURBT)	Intraoperative; vasopressor-requiring episodes	Smaller increase in MAP from awakening to anesthesia induction (<+6.5 mmHg); advanced age; general anesthesia	Monitor peri-induction MAP trend; anticipate vasopressor requirement; consider anesthesia strategy and closer hemodynamic monitoring in high-risk patients
Hypotension/nausea-vomiting/photosensitivity/liver dysfunction	Okabe et al., 2024 [56]	Hypotension: SBP < 80 mmHg requiring leg elevation or intravenous fluids, or continuous vasopressor infusion (perioperative).	Hypotension 12/104 (11.5%); nausea/vomiting 39/104 (37.5%); photosensitivity 33/104 (31.7%); liver dysfunction 54/104 (51.9%)	Not detailed; emphasizes perioperative vigilance and post-op precautions	Spinal anesthesia linked to hypotension; older age and higher BMI linked to nausea/vomiting; highest-risk profile: older obese men on RAS inhibitors.	Close BP monitoring (especially with spinal anesthesia); review antihypertensives including RAS inhibitors; baseline and selective post-op LFTs for high-risk patients; strict light-avoidance counseling with written instructions.
Hypotension/liver injury	Matsushita et al., 2025 [57]	AST/ALT elevation (CTCAE grade ≥ 3); hypotension (SBP < 80 mmHg); severe hypotension (continuous vasopressor)	Elderly (>70 years): AST/ALT grade ≥ 3 8/112 (7.1%) vs. 1/194 (0.5%); hypotension 84/112 (75.0%) vs. 111/194 (57.2%); severe hypotension 18/112 (16.1%) vs. 17/194 (8.8%)	Higher risks of liver injury and intraoperative hypotension in PDD group	Not detailed in abstract	Weigh benefit vs. risk in elderly; consider closer perioperative lab and hemodynamic monitoring
Hypotension	Hori et al., 2025 [61]	Moderate or severe intraoperative hypotension (as defined in the study)	10/26 (38%) (interval ≥ 4 h) vs. 54/88 (61%) (interval < 4 h)	Intraoperative; vasopressor requirement lower with ≥4 h interval	Intake-to-induction interval (<4 h vs. ≥4 h); other covariates as assessed in the study	Scheduling to extend the intake-to-induction interval (preferably ≥4 h) in higher-risk patients
Hypotension/nausea-vomiting	Hirao et al., 2026 [58]	Hypotension: SBP ≤ 80 mmHg (spinal anesthesia)	Hypotension 64/109 (58.7%); nausea/vomiting 29/109 (26.6%)	No Clavien–Dindo ≥3 events; generally manageable	No strong patient predictors for hypotension; phenylephrine use associated with nausea/vomiting (OR 10.2)	Treat hypotension pharmacologically as needed; anticipate N/V when vasopressors used; consider antiemetic strategy
Hepatic enzyme elevation	Taoka et al., 2026 (SPP2C102) [62]	AST/ALT and/or GGT elevation (CTCAE v5.0)	28/145 (19.3%)	All grade 1–2; transient	SBP ≥ 130 mmHg associated after adjustment	Baseline and targeted post-op LFTs for higher risk; generally self-limited; counsel patients
Nausea/vomiting	Taoka et al., 2026 (SPP2C102) [62]	CTCAE v5.0	28/145 (19.3%)	All grade 1–2	Female sex associated; DBP ≥ 80 mmHg borderline; prophylactic antiemetics numerically reduced rates	Selective prophylactic antiemetics; outpatient symptom instructions

Abbreviations: AE, adverse event; BP, blood pressure; SBP, systolic blood pressure; DBP, diastolic blood pressure; MAP, mean arterial pressure; BMI, body mass index; RAS, renin-angiotensin system; OR, odds ratio; WL, white light; LFT, liver function test; AST, aspartate aminotransferase; ALT, alanine aminotransferase; GGT, gamma-glutamyltransferase; CTCAE, Common Terminology Criteria for Adverse Events; TURBT, transurethral resection of bladder tumor.

**Table 3:** Comparative context of 5-ALA-based fluorescence guidance and other adjunct optical imaging modalities in urologic oncology.

Modality/Platform	Main Urologic Task	Representative Performance/Evidence	Main Strengths	Main Limitations/Translational Caveat
5-ALA–PDD (oral/intravesical; blue-light)	Wide-field lesion detection and resection guidance in NMIBC; emerging ureteroscopic use in UTUC	Oral 5-ALA phase III NMIBC: sensitivity 79.6% vs. 54.1% with WL; specificity 80.6% vs. 95.5% [14]	Strong fluorescence contrast for CIS/flat and multifocal lesions; most mature therapeutic-outcome data in urothelial disease	False positives with inflammation, recent TURBT, or intravesical therapy; oral regimens may cause hypotension or photosensitivity
HAL blue-light cystoscopy	Intravesical PDD for NMIBC/CIS	CIS detection 92% vs. 68% with WL; 9-month recurrence 47% vs. 56% in an RCT [26,28]	Largest multinational evidence-based; avoids systemic oral 5-ALA exposure	Requires intravesical instillation and dwell time; false-positive biopsies still occur in inflamed or recently treated bladder
NBI	Drug-free cystoscopic contrast enhancement in NMIBC	Head-to-head study: overall sensitivity 76.2% for NBI vs. 89.6% for PDD; specificity 46.3% for NBI vs. 22.5% for PDD; CIS sensitivity 54.1% for NBI vs. 94.6% for PDD [51]	No photosensitizer; immediate activation; convenient office and operating-room workflow	Enhances vascular pattern rather than tumor-specific fluorescence; performance for flat/CIS lesions may be less favorable than PDD in some series
pCLE	Real-time microscopic characterization/optical biopsy of suspicious bladder lesions	Prospective TURBT study: malignant lesion sensitivity 91.7%, PPV 93.6%; CIS vs. inflammation sensitivity 71.4%, specificity 81.3% [87]	Provides in vivo histology-like information; may support grading and biopsy targeting	Contact-based, small field of view, requires fluorescein and operator expertise; adjunct after lesion identification rather than a survey tool
Indocyanine green near-infrared fluorescence (ICG-NIRF)	Vascular/perfusion guidance and anatomic assistance during partial nephrectomy	In 100 RAPN cases, hypofluorescence for malignancy had PPV 87%, sensitivity 84%, specificity 57%, and NPV 52%; malignancy prediction remained unreliable [76]	Strong robotic integration; useful for perfusion assessment, selective clamping, and vascular anatomy	Not tumor-specific; limited histologic discrimination; complements rather than replaces tumor-directed fluorescence

Direct numerical comparisons across modalities should be interpreted cautiously because clinical indications, reference standards, and endpoints differ (e.g., lesion detection, microscopic characterization, or perfusion guidance).

## Data Availability

No new data were created or analyzed in this study. Data sharing is not applicable to this article.

## Abbreviations

The following abbreviations are used in this manuscript:

5-ALA	5-aminolevulinic acid
ABCG2	ATP-binding cassette sub-family G member 2
ACEi/ARB	angiotensin-converting enzyme inhibitor/angiotensin receptor blocker
AE	adverse event
AI	artificial intelligence
ALT	alanine aminotransferase
AST	aspartate aminotransferase
AUA	American Urological Association
AUA/SUO	American Urological Association/Society of Urologic Oncology
BCG	bacillus Calmette-Guérin
BLC	blue light cystoscopy
BLFC	blue light flexible cystoscopy
BMI	body mass index
BP	blood pressure
CIS	carcinoma in situ
CTCAE	common terminology criteria for adverse events
DBP	diastolic blood pressure
EAU	European Association of Urology
FECH	ferrochelatase
FPR	false-positive rate
GGT	gamma-glutamyltransferase
HAL	hexaminolevulinate
HR	hazard ratio
ICG	indocyanine green
ICG-NIRF	indocyanine green near-infrared fluorescence
LFTs	liver function tests
LPN	laparoscopic partial nephrectomy
MAP	mean arterial pressure
NBI	narrow-band imaging
NIRF	near-infrared fluorescence
NMIBC	non-muscle-invasive bladder cancer
NPV	negative predictive value
OR	odds ratio
pCLE	probe-based confocal laser endomicroscopy
PDD	photodynamic diagnosis
PDD-URS	photodynamic diagnosis ureterorenoscopy
PEPT1	peptide transporter 1
PpIX	protoporphyrin IX
PPV	positive predictive value
PSM	propensity score matching
PSMs	positive surgical margins
QALY	quality-adjusted life year
RALP	robot-assisted laparoscopic radical prostatectomy
RAPN	robot-assisted partial nephrectomy
RAS	renin–angiotensin system
RCC	renal cell carcinoma
RCT	randomized controlled trial
RD	risk difference
RFS	recurrence-free survival
RR	risk ratio
RRR	relative risk reduction
SAE	serious adverse event
SBP	systolic blood pressure
TURBT	transurethral resection of bladder tumor
URS	ureterorenoscopy
UTUC	upper tract urothelial carcinoma
WL	white light
WLC	white light cystoscopy
